# Pulsed Field Versus Radiofrequency Ablation in Concomitant Pulmonary Vein Isolation and LAA Occlusion: Impact on Peridevice Leak and Procedural Efficiency

**DOI:** 10.1111/jce.70286

**Published:** 2026-02-22

**Authors:** Mohammad Montaser Atasi, Shea Hubbard, Charles Campbell, Charbel Noujaim, Christian M. Massad, Michel Abou Khalil, Yara Menassa, Alex El Darzi, Maximilian Moersdorf, Carlo El Khoury, Chanho Lim, Yingshuo Liu, Yishi Jia, Abboud Hassan, Mayana Bsoul, Ghassan Bidaoui, Hadi Younes, Monique Young, Bassam Wanna, Amitabh C. Pandey, Omar Kreidieh, Han Feng, Qussay Marashly, Nassir F. Marrouche

**Affiliations:** ^1^ Tulane Research Innovation for Arrhythmia Discovery Tulane University New Orleans Louisiana USA

**Keywords:** atrial fibrillation, left atrial appendage occlusion, peridevice leak, pulmonary vein isolation, pulsed field ablation, radiofrequency ablation

## Abstract

**Background:**

Concomitant pulmonary vein isolation (PVI) and left atrial appendage occlusion (LAAO) is increasingly performed in patients with atrial fibrillation (AF) at elevated thromboembolic risk. Radiofrequency ablation (RFA) has been the conventional approach, but pulsed field ablation (PFA) may reduce tissue edema, procedural duration, and peridevice leak (PDL).

**Objective:**

To compare safety, efficacy, and PDL rates between PFA + LAAO and RFA + LAAO.

**Methods:**

In this single‐center, retrospective study, 175 consecutive patients undergoing LAAO with or without concomitant PVI between September 2021 and May 2025 were included: RFA + LAAO (*n* = 61), PFA + LAAO (*n* = 39), and LAAO‐only (*n* = 75). Procedural data, device characteristics, and complications were collected. The primary endpoint was PDL at 45‐day follow‐up transesophageal echocardiography (TEE). Secondary endpoints included procedure duration, vascular and esophageal complications, device‐related thrombosis, and arrhythmia recurrence.

**Results:**

Baseline characteristics were generally balanced, although RFA + LAAO patients were younger. No large PDL (> 5 mm) was observed. At 45 days, small PDL (≤ 5 mm) was significantly higher in RFA + LAAO (23%) vs. PFA + LAAO (5.1%) and LAAO‐only (9.3%; *p* = 0.015). Mean procedure duration was shorter with PFA + LAAO (67.1 ± 10.4 min) compared with RFA + LAAO (99.6 ± 12.8 min; *p* < 0.001). Major complications and device‐related thrombosis were rare. Minor complications and AF recurrence were comparable across groups.

**Conclusions:**

Concomitant PFA + LAAO is associated with lower PDL incidence and shorter procedural times compared with RFA + LAAO, without compromising safety.

**Abbreviations**
AF: atrial fibrillation
DOAC: direct oral anticoagulant
DRT: device‐related thrombosis
ICE: intracardiac echocardiography
LA: left atrium
LAA: left atrial appendage
LAAO: left atrial appendage occlusion
PDL: peri‐device leak
PFA: pulsed field ablation
PVI: pulmonary vein isolation
RFA: radiofrequency ablation
TEE: transesophageal echocardiography
VKA: vitamin K antagonist

## 1 Introduction

Atrial fibrillation (AF) is the most prevalent clinical arrhythmia and is associated with a significantly increased risk for thromboembolic events, estimated at approximately 5% annually, and reaching up to 15% in high‐risk patients. Although Vitamin K antagonists (VKA) and direct oral anticoagulants (DOAC) are highly effective in stroke prevention in AF, their use carries risks of major bleeding, suboptimal adherence, and dietary or drug–drug interactions [1]. Left Atrial Appendage Occlusion (LAAO) is a mechanical alternative to long‐term anticoagulation and has been shown to be non‐inferior to DOACs and VKA in preventing stroke and major bleeding events in appropriately selected patients [2, 3, 4]. Pulmonary vein isolation (PVI) via catheter ablation is the cornerstone for rhythm control therapy for AF [5]. However, risk of arrhythmia recurrence is established and carries a risk for stroke. Thus, patients continue to require long‐term anticoagulation after AF ablation [6]. Performing concomitant AF ablation and LAAO in a single procedure has been shown to be safe and effective [7, 8, 9, 10]. However, concerns have been raised regarding the risk of peri‐device leak (PDL) with concomitant procedures [11, 12, 13, 14].

## 2 Methods

### 2.1 Study Design and Patient Population

The aim of this single‐center, retrospective study is to evaluate the safety and efficacy of concomitant PFA and LAAO compared with concomitant RFA and LAAO.

Patient data were obtained from the research registry at the Tulane Research Innovation for Arrhythmia Discovery (TRIAD), Tulane University, New Orleans, Louisiana.

All concomitant procedures and LAAO procedures between September of 2021 and May of 2025 were reviewed.

Patients were enrolled consecutively. Inclusion criteria consisted of patients with atrial fibrillation with 2 or more risk factors for stroke and who received an LAAO, with or without concomitant ablation. Patients were excluded if they had prior LAAO, incomplete procedural data, or if follow‐up imaging data were unavailable. A favorable anatomy for undergoing LAAO was confirmed with Transesophageal Echocardiography (TEE).

### 2.2 RFA Procedure

All procedures were performed under general anesthesia with continuous hemodynamic and electrocardiographic monitoring. Ultrasound‐guided femoral venous access was achieved using the modified Seldinger technique. Intracardiac echocardiography (ICE) was used to delineate cardiac anatomy and exclude pericardial effusion. Transseptal access was performed using the VersaCross dedicated radiofrequency transseptal wire. The PENTARAY high‐density mapping catheter was used for LA mapping. Three‐dimensional mapping was performed using the CARTO electroanatomic mapping system (Biosense Webster). PVI was performed using a circumferential, continuous point‐by‐point lesion set (50 W) encircling each ipsilateral pulmonary vein pair at the antral level. Pulmonary vein entrance and exit block were then confirmed. In a subset of patients, additional lesion sets were delivered at the operator's discretion to further modify arrhythmogenic substrate; these cases were categorized as PVI +. Intraoperative anticoagulation with intravenous heparin was administered according to standard protocol to maintain the target activated clotting time throughout the procedure.

### 2.3 PFA Procedure

PFA was performed using the FARAPULSE PFA system (Boston Scientific, MA, USA). Ultrasound‐guided femoral venous access was achieved using the modified Seldinger technique. ICE was used to delineate cardiac anatomy and exclude pericardial effusion). Transseptal access was performed using the VersaCross dedicated radiofrequency transseptal wire. The FARAWAVE PFA catheter was advanced into the LA through the FARADRIVE steerable sheath. In the PFA group, all pulmonary veins were successfully isolated at the antral level using the catheter in “basket” and “flower” configurations. In selected patients, the operator performed additional lesion sets beyond PVI to target suspected mechanisms or atrial substrate contributing to AF; these cases were categorized as PVI +. Three‐dimensional mapping was used with the St Jude ESI, CARTO or Faraview electroanatomic mapping systems. Heparin dosing was used throughout the procedure to ensure an active clotting time (ACT) of greater than 300 s.

### 2.4 LAAO Procedure

Following completion of the ablation procedure, patients in the concomitant procedure groups underwent immediate LAAO with the WATCHMAN FLX or WATCHMAN FLX PRO devices (Boston Scientific, MA, USA). An access sheath and catheter were inserted via the previous transeptal puncture into the left atrium. The dimensions of the LAA and the ostium were confirmed with ICE and the appropriately sized Watchman device was selected. Device implantation and release within the ostium of the LAA was performed under ICE guidance. Intraoperative TEE was used to confirm good positioning and seal, and the device was released only if PASS criteria were met. Repeat ICE was performed to ascertain the absence of pericardial effusion, catheters and sheaths were removed, and hemostasis was achieved per the physician's standards.

### 2.5 Post Procedure Follow Up and Data Collection

The following procedural parameters were collected: device size, left atrial appendage ostium size, degree of device compression, and presence of PDL intraoperatively. After ablation and LAAO, patients received oral anticoagulants for at least 6 weeks under physician supervision until the absence of PDL was confirmed on 45‐day follow up TEE, then switched to Aspirin alone. The TEE was performed using multiplane imaging (0°, 45°, 90°, and 135° views) to assess device position, anchoring stability, and seal/peridevice leak prior to clinical decision‐making. The timing of the follow‐up TEE varied because scheduling depended on patient adherence and availability, resulting in some studies being performed earlier or later than the nominal 45‐day window. Safety endpoints included major procedural complications, vascular access complications, hospital length of stay, and arrhythmias incidence.

### 2.6 Statistical Analysis

Demographics and outcomes were summarized and compared among groups. Continuous variables were summarized via mean ± standard deviation (SD) and compared among groups through Wilcoxon tests or *t*‐tests, depending on the normality assumption check based on Shapiro–Wilk tests. Categorical variables were compared using *χ*² tests; Fisher's exact test was used when expected cell counts were < 5. A two‐sided significance level of 0.05 was considered. All the analyses were conducted by R (version 4.4.2).

### 2.7 Ethical Approval

This study was conducted using the Tulane Research Innovation Arrhythmia Discovery (TRIAD) Database. The protocol was reviewed and approved by the Tulane University Biomedical Institutional Review Board (IRB), administered through the Tulane University Human Research Protection Office (HRPO) (Study Number: 2019‐1803‐TUHSC). The study was performed in accordance with applicable institutional requirements and the ethical principles outlined in the Declaration of Helsinki.

## 3 Results

### 3.1 Patient Cohorts and Baseline Characteristics

Between September 2021 and June 2025, 175 patients underwent LAAO with or without concomitant pulmonary vein isolation: 61 underwent RFA‐PVI + LAAO, 39 underwent PFA + LAAO, and 75 underwent LAAO‐only. Baseline demographic and clinical characteristics are summarized in Table 1. Patients in the RFA‐PVI + LAAO group were younger (mean 69.98 ± 10.96 years) compared with the LAAO‐only (77.37 ± 6.48 years) and PFA‐PVI + LAAO (76.32 ± 8.63 years) groups (*p* < 0.001). The mean CHA₂DS₂‐VASc score was lower in RFA + LAAO 3.5 vs. LAAO‐only 4 and PFA + LAAO 4.2. Vascular disease was more prevalent in PFA + LAAO (28.2%) compared with LAAO‐only (17.3%) and RF + LAAO (6.6%; *p* = 0.014). No significant intergroup differences were observed in sex distribution, race, hypertension, diabetes mellitus, prior stroke/TIA or AF type (all *p* > 0.05). Additional lesion sets (PVI +) were more frequent in procedure PFA + LAAO than in RF + LAAO (66.7% vs. 39.3%; *p* = 0.007). Left atrial appendage closure device differed significantly. WATCHMAN FLX PRO was used in 92.3% of the patients in the PFA‐PVI + LAAO group vs. 16% in LAAO‐only and 11.5% in the RFA‐PVI + LAAO groups.

**Table 1:** Baseline characteristics.

Characteristic	RFA+LAAO (*n *= 61)	LAAO only (*n *= 75)	PFA+LAAO (*n *= 39)	*p* value
Age, years (mean ± SD)	69.98 ± 10.96	77.37 ± 6.48	76.32 ± 8.63	< 0.001
Male sex, *n* (%)	34 (55.7)	50 (66.7)	21 (53.8)	0.291
Race, *n* (%)				0.270
White	43 (70.5)	48 (64)	33 (84.6)	
African American	9 (14.8)	12 (16)	3 (7.7)	
Hispanic	8 (13.1)	15 (20)	3 (7.7)	
Asian	1 (1.6)	0 (0)	0 (0)	
CHA₂DS₂‐VASc score (mean ± SD)	3.53 ± 1.38	4 ± 1.50	4.21 ± 1.51	0.093
CHF, *n* (%)	11 (18.0)	17 (22.7)	9 (23.1)	0.761
Hypertension, *n* (%)	50 (82.0)	61 (81.3)	30 (76.9)	0.804
Diabetes, *n* (%)	17 (27.9)	22 (29.3)	6 (15.4)	0.241
Stroke/TIA, *n* (%)	7 (11.5)	10 (13.3)	9 (23.1)	0.250
Vascular disease, *n* (%)	4 (6.6)	13 (17.3)	11 (28.2)	0.014
AF type, *n* (%)				0.470
Paroxysmal	37 (60.7)	44 (58.7)	19 (48.7)	
Persistent	24 (39.3)	31 (41.3)	20 (51.3)	
Ablation strategy, *n* (%)				0.007
PVI‐only	37 (60.7)	—	13 (33.3)	
PVI+	24 (39.3)	—	26 (66.7)	
Closure device type, *n* (%)				< 0.001
WATCHMAN FLX PRO	7 (11.5)	12 (16)	36 (92.3)	
WATCHMAN FLX	54 (88.5)	63 (84)	3 (7.7)	

*Note:* Values are mean ± SD or *n* (%).

Abbreviations: CHF = congestive heart failure, PFA = pulsed field ablation, PVI = pulmonary vein isolation, PVI+ = pulmonary vein isolation plus additional ablation, RF = radiofrequency, TIA = transient ischemic attack, WM = Watchman.

### 3.2 Procedural Outcomes and Safety

**Figure 1:**
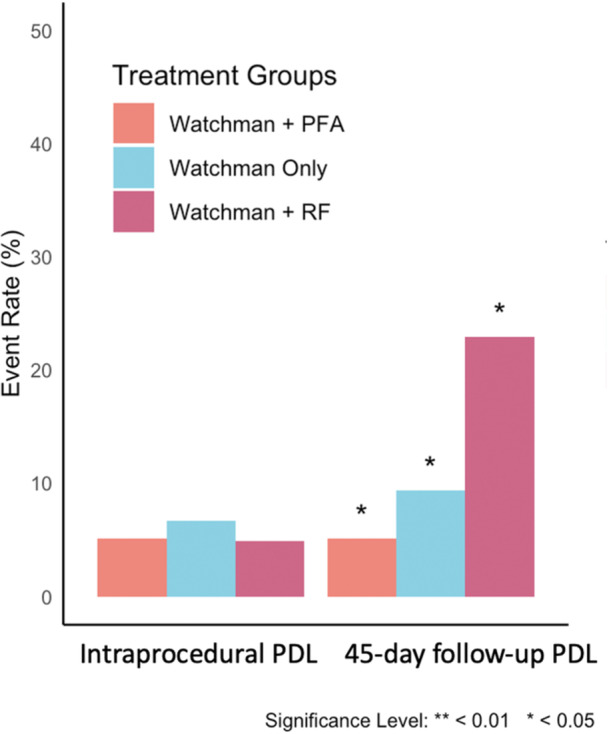
Incidence of minor peridevice leak intraprocedurally and at 45‐day follow‐up TEE Rates of minor PDL (≤ 5 mm) are shown across groups undergoing PFA + Watchman, Watchman only, and RFA + Watchman. Intraprocedural PDL incidence was low and comparable among groups. At 45‐day follow‐up, PDL was significantly higher in the RFA group compared with PFA and Watchman‐only groups (*p* < 0.013). PDL, peridevice leak; PFA, pulsed field ablation; RFA, radiofrequency ablation.

Procedural outcomes and complications are detailed in Table 2 and Figure 1. No large PDL (> 5 mm) were observed. Small intraprocedural PDL (≤ 5 mm) were rare and did not differ significantly among groups. Intraprocedural device compression rates were similar across groups.

**Table 2:** Incidences of peridevice leak and complications.

Outcome	RFA+LAAO (*n *= 61)	LAAO only (*n* = 75)	PFA+LAAO (*n *= 39)	*p* value
Large peridevice leak (> 5mm), *n* (%)				
Intraprocedural	0 (0.0)	0 (0.0)	0 (0.0)	
Follow‐up	0 (0.0)	0 (0.0)	0 (0.0)	
Small peridevice leak (≤ 5mm), *n* (%)				
Intraprocedural	3 (4.9)	5 (6.7)	2 (5.1)	0.894
45‐day follow‐up	14 (23.3)	7 (9.3)	2 (5.1)	0.015
Compression rate, % (Mean ± SD)	28.4 ± 10.5	31.2 ± 17.8	27.7 ± 13.9	0.88
Time from procedure to follow‐up TEE Mean (Min‐Max)	103.5 (41–461)	116.4 (18–735)	49.6 (14–124)	0.27
Major access site complications, *n* (%)				
Arteriovenous fistula	0 (0.0)	0 (0.0)	0 (0.0)	
Arterial pseudoaneurysm	1 (1.6)	0 (0.0)	0 (0.0)	0.390
Minor access site complications, *n* (%)				
Bleeding	1 (1.6)	4 (5.3)	1 (2.6)	0.472
Hematoma	4 (6.6)	1 (1.3)	1 (2.6)	0.236
Esophageal ulcer, *n* (%)	2 (3.3)	0 (0.0)	0 (0.0)	0.151
Device‐related thrombosis, *n* (%)	1 (1.6)	1 (1.3)	0 (0.0)	0.737
AF recurrence, *n* (%)	14 (29.8)	—	8 (20.5)	0.326
Follow‐up time, Mean (Min‐Max)	374 (281–533)	—	167 (61–318)	< 0.001
Procedure time (Mean ± SD)	99.6 ± 12.8	48.8 ± 9.5	67.1 ± 10.4	< 0.001

*Note:* Includes access‐site bleeding, hematoma, and pseudoaneurysm.

Abbreviations: LAAO = left atrial appendage occlusion, PDL = peridevice leak, PFA = pulsed‐field ablation, RF = radiofrequency.

**Figure 2:**
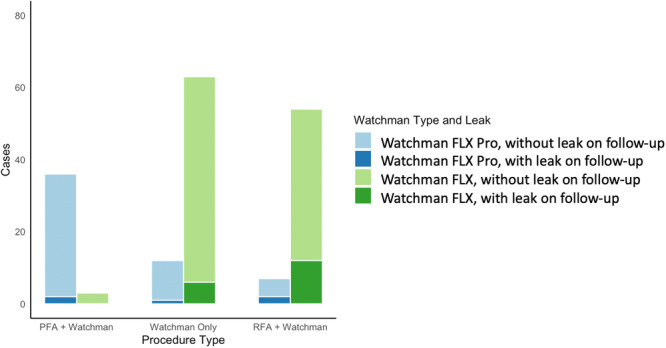
Observed 45‐day PDL incidence according to Watchman device type within each study group PDL was observed less frequently in the PFA + Watchman group compared with the RFA + Watchman group, with the lowest incidence seen in patients receiving Watchman FLX Pro. Stratification of PDL by device type revealed no statistically significant difference (9.09% for Watchman FLX Pro vs. 15% for Watchman FLX, *p* = 0.282). PDL, peridevice leak; PFA, pulsed field ablation; RFA, radiofrequency ablation.

At 45‐day follow‐up, minor PDL incidence was significantly higher in RFA‐PVI + LAAO group (23.3%) compared with LAAO‐only (9.3%) or PFA‐PVI + LAAO (5.1%) groups (*p* = 0.013). In a sensitivity analysis, 45‐day PDL was not associated with ablation extent; in the PVI‐PFA + LAAO group (*n* = 39), PDL occurred in 1/13 (7.7%) with PVI‐only vs. 1/26 (3.8%) with PVI+ (*p* = 0.61). In the RFA + LAAO group (*n* = 61), PDL occurred in 9/37 (24.3%) with PVI‐only vs. 5/24 (20.8%) with PVI+ (*p* = 0.75). Stratification of PDL by device type revealed no statistically significant difference (9.09% for WATCHMAN FLX PRO vs. 15% for WATCHMAN FLX, *p* = 0.282) (Figure 2).

**Figure 3:**
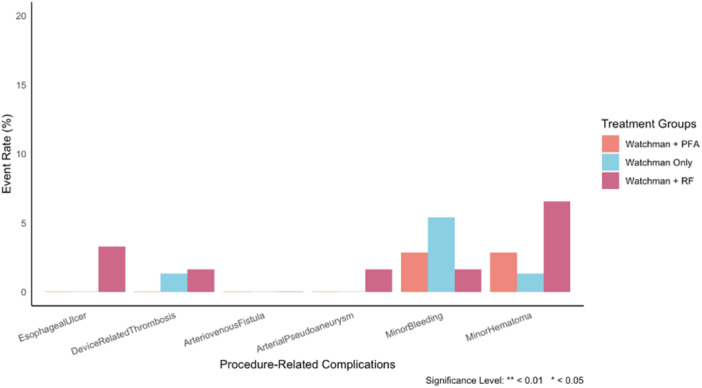
Incidence of procedure‐related complications across study groups Procedure‐related complications were infrequent and nonsignificant across all groups. Esophageal ulceration observed only in the RFA group. Minor access‐site events occurred more commonly with RFA compared with PFA and Watchman‐only. PFA, pulsed field ablation; RFA, radiofrequency ablation.

Major vascular access site complications were rare and not statistically significant among all groups (Figure 3). One case of arterial pseudoaneurysm occurred in the LAAO‐only group (1.6%).

Minor access site complications were infrequent. In the RF + LAAO group, minor complications occurred in 5 patients (1.6% bleeding, 6.6% hematoma). The LAAO‐only group had 5 minor complications (5.3% bleeding, 1.3% hematoma), and the PFA + LAAO group, had two (2.6% bleeding and 2.6% hematoma). There were no significant differences in the rates of bleeding (*p* = 0.472) or hematoma (*p* = 0.236) between groups. Esophageal ulceration was observed in 2 patients (3.3%) in the RF + LAAO group, with no cases reported in the other groups. This difference was not statistically significant (*p* = 0.151). Device‐related thrombosis was rare, occurring in only one patient (1.6%) in the RF + LAAO group, and one (1.33%) in the LAAO‐only group (Figure 3). AF recurrence was similar between RF + LAAO and LAAO‐only (29.8% vs. 20.5%; *p* = 0.326) during the follow‐up period of 374 and 167 days respectively.

**Figure 4:**
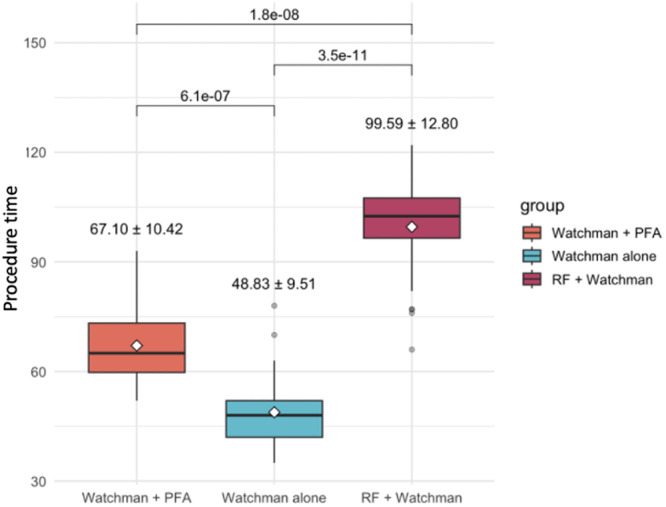
Comparison of procedural time across the study groups. Mean procedure duration was significantly shorter with PFA + Watchman (67.1 ± 10.4 min) compared with RFA + Watchman (99.6 ± 12.8 min, *p* < 0.001). Watchman‐only procedures had the shortest procedural duration.

Procedure duration was significantly longer in the RFA‐PVI + LAAO group (99.6 min) compared with LAAO‐only (48.8 min) and PFA‐PVI + LAAO (67.1 min) groups (*p* < 0.001) (Figure 4).

## 4 Discussion

The major findings of this study are: (i) compared with concomitant RFA, the use of PFA in conjunction with LAAO was associated with a significantly lower incidence of minor PDL at 45‐day follow‐up; and (ii) the PFA approach was associated with significantly reduced procedure duration while maintaining a comparable safety profile.

### 4.1 PDL Incidence

Although the RFA + LAAO and PFA + LAAO groups exhibited similar rates of intraprocedural PDL, the RFA group experienced a significant increase from 4.9% intraprocedurally to 23% at 45‐day follow‐up. In contrast, the incidence of PDL remained stable in both the PFA + LAAO and standalone LAAO groups (Table 2 and Figure 1).

The higher incidence of new PDL observed with RFA concomitant procedures aligns with prior studies. Zhu et al. reported that despite a similar intraprocedural PDL incidence, a significantly greater rate of new PDL at 6 weeks in the combined RFA + LAAO group compared to standalone LAAO (25.5% vs. 8.5%; *p* = 0.03) [9]. Similarly, Wintgens et al. demonstrated a PDL incidence of 7.4% intraprocedurally and 28.6% at 3‐month follow‐up after RFA concomitant procedures [15].

Despite advances in device design that have reduced PDL incidence, rates remain clinically significant. Data from the PINNACLE FLX randomized trial and the SURPASS registry demonstrated a PDL incidence of 17.4% and 18% at 45‐day follow‐up with Watchman FLX devices [16, 17]. PDLs primarily result from incomplete sealing and inadequate endothelialization and are associated with increased risk of thromboembolic events [18, 19, 20] Consequently, clinically significant PDLs often necessitate long‐term anticoagulation and potentially further invasive treatment options [20]. On the other hand, minor PDLs (≤ 5 mm) were historically considered an acceptable residual jet; in PROTECT AF, discontinuation of warfarin at 45 days was allowed when the peri‐device leak was ≤ 5 mm on TEE [21]. More recent data, however, suggest that even small leaks (0–5 mm)—especially when they persist on 1‐year follow‐up TEE (~55%)—may be associated with a modestly higher long‐term thromboembolic risk [18, 19, 22]. Therefore, minor PDLs do not uniformly require prolonged anticoagulation, but they should prompt follow‐up imaging and individualized antithrombotic management based on the patient's thromboembolic and bleeding risk.

Recent research has quantified the edema occurring in the left atrial ridge following ablation, which can complicate accurate device sizing, reduce compression rate over time, and contribute to PDL as the edema resolves [23]. PFA has been shown to be non‐inferior to RFA and cryoballoon ablation and has been associated with reduced acute tissue inflammation and edema [10, 24]. Nakatani et al. reported that PFA, in contrast to RFA, is associated with less edema formation [10]. This may explain the lower incidence of PDL 45 days after concomitant PF ablation and LAAO procedures (Central Illustration 1).

**Central Illustration 1:**
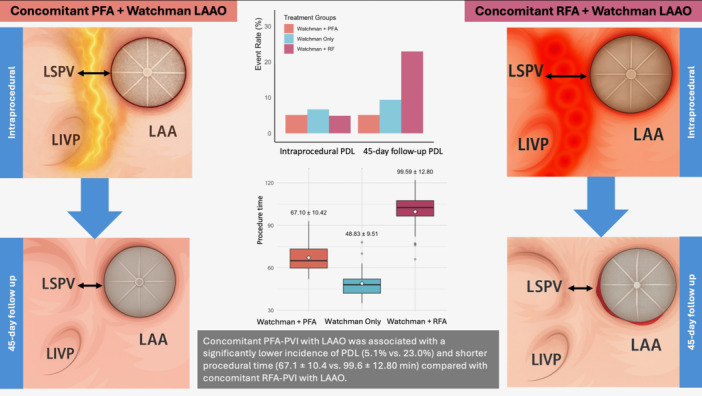
Concomitant PFA‐PVI and LAAO: Impact on Peridevice Leak and Procedural Time Concomitant PFA + Watchman was associated with a significantly lower incidence of PDL at 45 days (5.1% vs. 23.3%) and shorter procedural duration (67.1 ± 10.4 min vs. 99.6 ± 12.8 min) compared with concomitant RFA + Watchman. The illustration highlights that RFA may induce tissue edema along the left atrial ridge (double‐arrow), which can resolve over time and contribute to device seal loss and PDL, whereas PFA produces less edema and a more durable seal. PDL, peridevice leak; PFA, pulsed field ablation; RFA, radiofrequency ablation.

### 4.2 Concomitant Procedure Duration

The combined PFA and LAAO procedure group exhibited shorter mean procedure times than the combination procedure using RFA. The mean procedure time for PFA + LAAO was 67.1 min compared to 99.6 min for the RFA + LAAO group. The group which underwent LAAO alone had a mean procedural time of 48.8 min (Figure 4 and Table 2).

Published data on concomitant RFA + LAAO procedures reported a mean procedural time of 177 min [25]. PFA has been shown to improve procedural duration compared to RFA [26, 27]. Beney et al. have reported a median procedure duration of 79 min (Range 60–120) with PFA concomitant procedures [28]. In addition to reduction in potential complications such as esophageal injury and phrenic nerve injury with PFA compared to RFA, improvement in procedural duration and time under general anesthesia can potentially improve procedural safety and has been suggested to correlate with better patients' perceived quality of life [29].

The impact of procedural duration and the combination of PFA with LAAO on long‐term clinical outcomes has not been extensively studied in large clinical trials or registries. We believe this represents an important area for future investigation, particularly when considering concomitant ablation and occlusion procedures.

### 4.3 Safety and Complications

Our study demonstrates that concomitant PVI using PFA with LAAO is feasible and exhibits a safety profile comparable to standalone LAAO and concomitant RFA with LAAO. Overall complication rates were low across cohorts. Notably, esophageal ulceration occurred only in the RFA + LAAO group (3.3%), while minor vascular complications were infrequent and similar across groups. Major vascular complications were absent in PFA + LAAO and standalone LAAO groups, with one arterial pseudoaneurysm reported in the RFA + LAAO cohort (1.6%) (Figure 3 and Table 2).

Device‐related thrombosis (DRT) remains a rare but important complication of LAAO. In our cohort, DRT incidence was low, with a single case in the standalone LAAO group and none in the combined ablation groups. This aligns with prior studies reporting decreasing DRT rates with improved procedural techniques and surveillance, typically below 5% and often resolved with anticoagulation [13, 23].

### 4.4 Study Limitations

This study was conducted at a single center with a modest sample size, particularly in the PFA + LAAO group, which limits statistical power and generalizability. The small sample size in the PFA group may have exaggerated the observed rate of vascular access complications and underpowered comparisons with the other groups. Baseline characteristics also differed between groups, with younger age and lower CHA₂DS₂‐VASc scores in the RFA + LAAO group, raising the possibility of residual confounding despite statistical comparisons; however, this is unlikely to be affecting the incidence of PDL. Follow‐up was limited to 45 days, which may not reflect the long‐term incidence of PDL, device‐related thrombosis, or clinical outcomes such as stroke and systemic embolism.

Procedural outcomes may have been influenced by operator experience and evolving practice over the study period, particularly given the novelty of PFA, which may limit the generalizability of data. Moreover, Device type was imbalanced across cohorts, with WATCHMAN FLX PRO used predominantly in the PFA + LAAO group. This nonrandom device selection could confound comparisons of PDL, as device design may influence sealing independent of ablation modality. Although stratified analyses by device type were not statistically significant, the limited sample size and event counts may have reduced power to detect device‐related differences. Finally, while the findings suggest shorter procedural times and fewer esophageal complications with PFA, the study was not powered to assess differences in rare but clinically significant events or to evaluate long‐term anticoagulation discontinuation and stroke prevention efficacy.

## 5 Conclusion

This study demonstrates that concomitant PFA‐PVI combined with left atrial appendage occlusion LAAO is associated with a significantly lower incidence of PDL during short‐term follow‐up, as well as a significantly reduced procedure duration compared to concomitant RFA‐PVI with LAAO. Larger, multicenter randomized studies with longer follow‐up are needed to confirm these results and better define the role of combining PFA with LAAO.

## Author Contributions

All authors contributed to the review article conception, data collection, and writing.

## Funding

The authors received no specific funding for this work.

## Conflicts of Interest

The authors declare no conflicts of interest.
